# Neonatal resuscitation: Current issues

**DOI:** 10.4103/0019-5049.71042

**Published:** 2010

**Authors:** Indu A Chadha

**Affiliations:** Department of Anaesthesiology, B J Medical College, Ahmedabad - 38 0016, India

**Keywords:** Current issues, guidelines, neonatal resuscitation, resuscitation

## Abstract

The following guidelines are intended for practitioners responsible for resuscitating neonates. They apply primarily to neonates undergoing transition from intrauterine to extrauterine life. The updated guidelines on Neonatal Resuscitation have assimilated the latest evidence in neonatal resuscitation. Important changes with regard to the old guidelines and recommendations for daily practice are provided. Current controversial issues concerning neonatal resuscitation are reviewed and argued in the context of the ILCOR 2005 consensus.

## INTRODUCTION

Neonatal Asphyxia accounts for 20.9% of neonatal deaths. Although the vast majority of newly born infants (90%) do not require intervention to breathe during transition from intrauterine to extrauterine life, approximately 10% of the newborns require some assistance to begin breathing at birth, and about 1% require extensive resuscitative measures.[1–3]

The goals of neonatal resuscitation are to prevent the morbidity and mortality associated with hypoxic-ischaemic tissue (brain, heart, kidney) injury and also to re-establish adequate spontaneous respiration and cardiac output.[2,3]

Guidelines for neonatal resuscitation have been issued by the American Heart Association and the American Academy of Paediatrics. The guidelines are helpful in remembering the sequence for resuscitation. Failure to follow the guidelines has resulted in bad outcomes.[1,2]

A rapid assessment of newly born infants who do not require resuscitation can generally be identified by the following four characteristics:

1. Was the infant born after a full-term gestation?
2. Is the amniotic fluid clear of meconium and evidence of infection?
3. Is the infant breathing or crying?
4. Does the infant have good muscle tone?

If the answer to all four of these questions is ‘yes,’ the infant does not need resuscitation and should not be separated from the mother. The infant can be dried, placed directly on the mother’s chest and covered with dry linen, to maintain temperature. Observation of breathing, activity and colour should be ongoing.

If the answer to any of these assessment questions is ‘no,’ there is a general agreement that the infant should receive one or more of the following four categories of action in sequence:

1. Initial steps in stabilisation (provide warmth, position, clear airway, dry, stimulate, re-position)
2. Ventilation
3. Chest compressions
4. Administration of epinephrine and / or volume expansion

**Figure 1:**
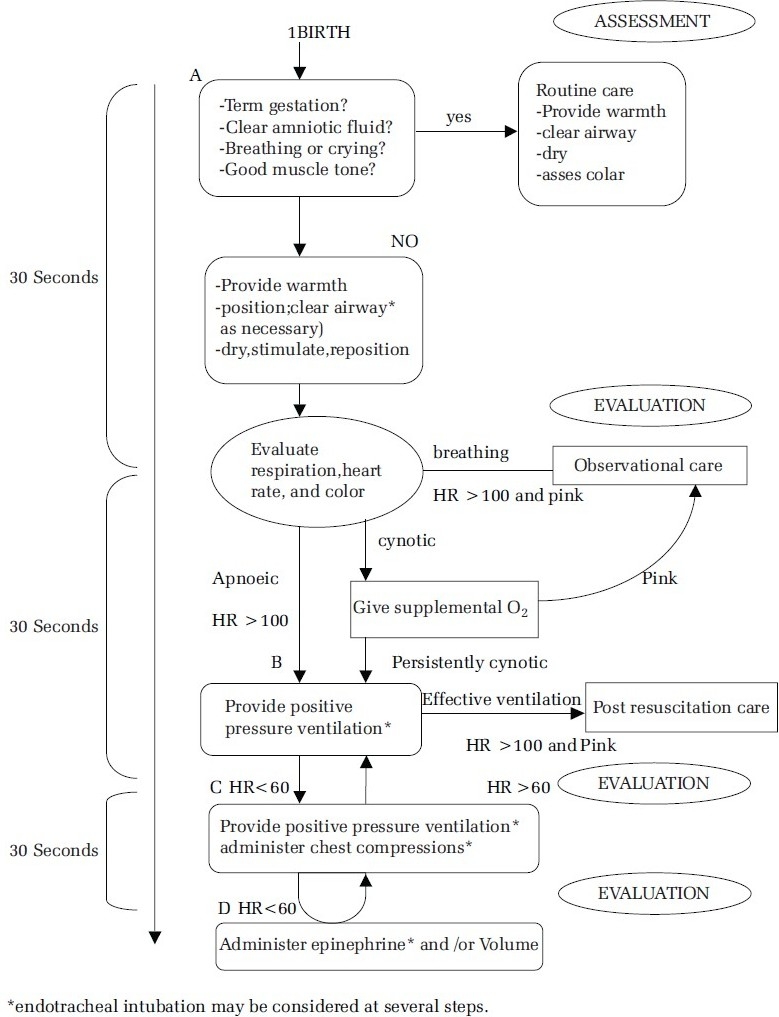
Neonatal flow algorithm (Neonatal Resuscitation Guidelines, Circulation, 2005)

The decision to progress from one category to the next is determined by the simultaneous assessment of three vital signs: respiration, heart rate and colour. Approximately 30 seconds is allotted to complete each step, re-evaluate and decide whether to progress to the next step[1–4] [Figure 1].

## INITIAL STEPS

The initial steps of resuscitation are to provide warmth by placing the infant under a radiant heat source, position the head in a ‘sniffing’ position to open the airway, clear the airway with a bulb syringe or suction catheter, dry the infant and stimulate breathing. Evaluation of the neonate for respiration, heart rate and colour at every 30-second interval must be done[1–7] [Figure 1].

During delivery, if the amniotic fluid is meconium stained:

- If the baby is vigorous (strong respiratory effort i.e., cry, good muscle tone, heart rate > 100 bpm) at birth, clear the airway by suctioning mouth first and then the nose with a bulb syringe or suction catheter. If bradycardia occurs during suctioning then stop suctioning and re-evaluate the heart rate. No intubation suctioning is required.
- If the baby is not vigorous (depressed respiration, depressed muscle tone and heart rate < 100 bpm), the newborn requires tracheal suctioning. First insert a laryngoscope and clear the mouth and posterior pharynx by using a suction catheter under direct vision, then insert the endotracheal tube into the trachea. Attach a suction device to the endotracheal tube. Apply suction as the tube is slowly withdrawn. Repeat if necessary till the meconium is recovered or until the heart rate indicates < 60 bpm, after which resuscitation must proceed without delay. A gentle but firm stimulation is given; gently flick the soles and rub the back.
- If heart rate is low, that is, < 100 bpm, positive pressure ventilation (PPV) should be provided without suctioning of the trachea.
- If the newborn is breathing and pink and has a heart rate >100 bpm, observe him.
- If the newborn is not breathing and remains apnoeic or gasping, has a heart rate of < 100 bpm or appears blue, the next step is to assist the neonate’s breathing by positive pressure ventilation, and if he is cyanotic supplemental oxygen is to be given.
- After about 30 seconds of ventilation and / or supplemental oxygen, evaluation is done again.
- If the newborn starts breathing, becomes pink and has a heart rate of > 100 bpm, post resuscitation care must be given.
- If heart rate is > 60 bpm, then support of the circulation by chest compression and positive pressure ventilation must be continued till the heart rate reaches > 100 bpm and the newborn becomes pink.
- If the heart rate is < 60 bpm, then support of the circulation by chest compression and positive pressure ventilation must be done. After about 30 seconds, evaluation is done again.
- If the heart rate is still < 60 bpm then epinephrine is administered along with continued PPV and chest compression. If the heart rate remains < 60 bpm, chest compression, positive pressure ventilation, and epinephrine can be repeated every three to five minutes.
- In case of placental abrupt, placenta previa or blood loss from the umbilical cord, the baby may not improve despite effective ventilation, chest compression and epinephrine. The baby will look pale, have delayed capillary refill, weak pulse and a low heart rate. The baby may be in hypovolemic shock and will need volume support.

The above guidelines are to be followed during every delivery of a newborn.

Although the Apgar score is a simple useful guide to neonatal well-being and resuscitation, it is only a guide. It is useful to convey the information about a newborn’s overall status and response to resuscitation at the time points, during resuscitation. The one minute score correlates well with acidosis and survival. The five minute score may or may not be predictive of the neurological outcome.[6,11]

In every delivery room, an area should be allotted for neonatal resuscitation, with all the necessary equipment and drugs stored nearby [Table 1]. During every delivery there should be at least one person whose primary responsibility is the new born. This person must be capable of initiating resuscitation, including administration of positive-pressure ventilation and chest compression.[1,3]

**Table 1:** Neonatal resuscitation supplies and equipment

1. Suction equipment
Bulb syringe Mechanical suction and tubing Suction catheters, 5F, 6F, 8F, 10F, 12F or 14F 8F feeding tube and 20 ml syringe Meconium aspirator
2. Bag and Mask equipment
Device for delivering positive pressure ventilation, capable of delivering 90 to 100% O_2_ Face masks – newborn and premature sizes (cushioned-rim masks preferred) O_2_ source with flowmeter (flow rate up to 10 l/min) and tubing
3. Intubation equipment
Laryngoscope with straight blades, No.0 (preterm) and No.1 (term) Extra bulbs and batteries for laryngoscope Endotracheal tubes 2.5,3.0,3.5,4.0-mm internal diameter (ID) Stylet (optional) Scissors Tape or securing device for ET tube Alcohol sponges CO_2_ detector or capnograph Laryngeal mask airway (optional)
4. Medication
Epinephrine 1:10,000(0.1 mg/ml) – 3 ml or 10 ml amp Isotonic crystalloids (normal saline or Ringer’s lactate) for volume expansion – 100 or 250 ml Sodium bicarbonate 4.2% (5 mEq/10 ml) – 10 ml amp Naloxone hydrochloride (0.4 mg/ml) – 1 ml amp or (1 mg/ml) 2 ml amp Dextrose 10% – 250 ml, normal saline for flushes Umbilical vessel catheterization supplies:- Sterile gloves, scalpel or scissors, antiseptic solution, umbilical tape, umbilical catheters, 3.5F, 5F, three-way stopcock Syringes 1, 3, 5, 10, 20, 50 ml needles 25, 21, 18G or puncture device for a needleless system
5. Miscellaneous
Gloves and appropriate personal protection Radiant warmer or other heat source Firm padded resuscitation surface Clock with seconds hand (timer optional) Warmed linens Stethoscope (neonatal head preferred) Tape half or three-fourth inches Cardiac monitor and electrodes or pulse oximeter and probe (optional for delivery room) Oropharyngeal airway (0, 00, and 000 sizes or 30, 40, 50 mm lengths)
6. For very pre-term babies
Compressed air source O_2_ blender to mix O_2_ and compressed air Pulse oximeter and probe Re-closeable, food-graded plastic bag (1-gallon size) or plastic wrap Chemically activated warming pad Transport incubator to maintain baby’s temperature during its move to the nursery

In high-risk births, the majority of newborns requiring resuscitation can be identified before birth. If need for resuscitation is anticipated, additional skilled personnel should be recruited and the necessary equipment prepared. A team of skilled personnel are required at the delivery of the baby — one for position suctioning and drying, and others for airway and endotracheal intubation, and a fourth for medication.[1–3] If a pre-term delivery (< 37 weeks of gestation) is expected, special preparations will be required.

## SPECIAL SITUATIONS

Conditions like Choanal atresia, pharyngeal airway malformations, laryngeal web, pneumothorax, plural effusion and Diaphramatic hernia should be looked for. Those requiring immediate interventions like putting an airway in the mouth for patency of neonates’ airway, by nasopharyngeal airway, tracheotomy or by insertion of intercostal drains must be done.[1–4]

## POST-RESUSCITATION CARE

Infants who require resuscitation are at risk of deterioration after their vital signs have returned to normal. Once adequate ventilation and circulation have been established, the infant should be maintained in or transferred to an environment in which close monitoring and anticipatory care can be provided.[1–4]

## GUIDELINES FOR WITHHOLDING AND DISCONTINUING RESUSCITATION

Morbidity and mortality for newborns vary according to the region and availability of resources.[2]

### 1. Withholding resuscitation

For conditions associated with high mortality and poor outcome, withholding resuscitative efforts may be considered, particularly when there has been parental agreement.[2,11,12] A consistent and coordinated approach to individual cases by the obstetric and neonatal teams and the parents is an important goal.[2,10,12]

Non-initiation of resuscitation and discontinuation of life-sustaining treatment during or after resuscitation are ethically equivalent, and clinicians should not hesitate to withdraw support with no functional survival. The following guidelines must be interpreted:[2,11]

1. When gestation, birth weight or congenital anomalies are associated with certain early death and unacceptably high morbidity, resuscitation is not indicated, for example, extreme prematurity (gestational age < 23 weeks or birth weight < 400 g), anencephaly or chromosomal abnormalities, such as trisomy 13.
2. In conditions with a high rate of survival and acceptable morbidity, resuscitation is nearly always indicated, for example, infant with gestational age 25 weeks and infant with congenital malformations.
3. In conditions associated with uncertain prognosis, wherein survival is borderline, the morbidity rate is high and the anticipated burden to the child is high, parental desires concerning initiation of resuscitation should be supported.

### 2. Discontinuing resuscitative efforts

Infants without signs of life (no heart beat and no respiratory effort) after 10 minutes of resuscitation show either a high mortality or severe neuro-developmental disability.[11–13] Therefore, after 10 minutes of continuous and adequate resuscitative efforts, discontinuation of resuscitation may be justified.[11–13]

## CURRENT ISSUES

### Temperature control

For the full-term newborn both standard thermal care (removing wet blankets, prompt drying, warming pads, wrapping the infant in a warm blanket, placing the infant skin-to-skin with the mother and covering both with a blanket) and placing the dried infant under a radiant heater are effective in maintaining normal body temperature.[14,15] Several trials have shown that, in addition to radiant heating, covering premature infants up to the neck in a transparent plastic wrapping (heat-resistant, food-grade) without previous drying, results in a higher body temperature of the newborn at admission, especially in infants < 28 weeks’ gestation.[14,16–19] Only the head is dried and covered with a cap. All resuscitation procedures, including intubation, chest compressions, and insertion of (central) lines, can be performed with the plastic cover in place. Currently, there is no evidence that this procedure improves mortality or the long-term outcome. Monitoring of body temperature should be considered, especially when resuscitation is prolonged, to avoid the small risk of inducing hyperthermia.[17,19,20]

Infants born to febrile mothers have been reported to have a higher incidence of perinatal respiratory depression, neonatal seizures, cerebral palsy and increased risk of mortality.[21–24] Hyperthermia should be avoided. The goal is to achieve normothermia and avoid iatrogenic hyperthermia.

### Clearing the airway of meconium

Aspiration of meconium before delivery, during birth or during resuscitation can cause severe meconium aspiration pneumonia (MAS) in 2 – 9% of the newborn infants.[25] One obstetrical technique, to try to decrease aspiration has been to suction the meconium from the infant’s airway after delivery of the head, but before delivery of the shoulders (intrapartum suctioning). Studies suggest that intrapartum suctioning may be effective for decreasing the risk of the aspiration syndrome,[26–28] but evidence from a large trial did not show such an effect.[29] Therefore, current recommendations no longer advise routine intrapartum oropharyngeal and nasopharyngeal suctioning. In the case of meconium-stained amniotic fluid and a non-vigorous newborn, endotracheal suction by brief intubation or suction under direct vision is advised. If the infant is vigorous, endotracheal suction is not recommended, because it may cause harm and does not improve the outcome.[30]

### Administration of oxygen

A normal newly born infant achieves and maintains pink mucous membranes without administration of supplementary oxygen.[31,32] Continuous oximetry has shown that neonatal transition is a gradual process.[31–33] Healthy term newborns reach pre-ductal oxygen saturations, between 79 and 91%, 5 minutes after birth,[34] and it may take > 10 minutes to achieve a pre-ductal oxygen saturation of > 95% and nearly one hour to achieve post-ductal saturation of > 95%.[35,36]

Newborns delivered by caesarean section and preterms reach average pre-ductal oxygen saturations of 90%, two minutes later than the healthy term newborns.[31,32] There is concern about the potential adverse effects of 100% oxygen on the respiratory physiology, cerebral circulation, and tissue damage from oxygen-free radicals. Conversely, there is also concern about tissue damage from oxygen deprivation during and after asphyxia. Studies on blood pressure, cerebral perfusion, and various biochemical measures of cell damage in asphyxiated animals, resuscitated with 100% oxygen versus 21% oxygen (room air), have shown conflicting results.[37–42] Study of preterm infants (< 33 weeks of gestation) exposed to 80% oxygen found lower cerebral blood flow when compared with those stabilized using 21% oxygen.[43] Meta-analysis studies showed a reduction in mortality rate and no harm in infants resuscitated in room air than with 100% oxygen.[44,45]

Supplementary oxygen is recommended whenever positive-pressure ventilation is indicated for resuscitation; free-flow oxygen should be administered to infants who are breathing, but have central cyanosis. The standard approach for resuscitation is to use100% oxygen. Some clinicians may begin resuscitation with an oxygen concentration of less than 100% and some may start with room air. Both these practices during resuscitation of neonates are reasonable. If the clinician begins resuscitation with room air, supplementary oxygen must be available to use if there is no appreciable improvement within 90 seconds of the birth. In situations where supplementary oxygen is not readily available, positive-pressure ventilation should be administered with room air. Administration of a variable concentration of oxygen guided by pulse oximetry may improve the ability to achieve normoxia more quickly.

#### Initial breaths and assisted ventilation

In term infants, initial inflations — either spontaneous or assisted — create a functional residual capacity.[46–50] The optimum pressure, inflation time, and flow rate required to establish an effective functional residual capacity have not been determined.

Inflation breaths are used in newborn resuscitation, to facilitate the aeration of the fluid-filled lungs, by applying a higher airway pressure for a prolonged period of time. When a pressure of 30 cm H_2_O is applied for a duration of five seconds, a higher lung volume is achieved than in the conventional one-second inflations.[47] One trial in preterm newborns has shown that sustained initial inflations through a nasopharyngeal tube, followed by nasal Continuous Positive airway pressure (CPAP), reduces the need for intubation.[47] Although the evidence is based on a few studies, inflation breaths may have a positive effect on postnatal adaptation for newborns in need of resuscitation.

Usually, the average initial peak inflating pressures of 30 to 40 cm H_2_O successfully ventilate unresponsive term infants.[46,48,49–51] Assisted ventilation rates of 40 to 60 breaths per minute are commonly used, but the relative efficacy of various rates has not been investigated.

The primary measure of adequate initial ventilation is the prompt improvement in heart rate. Chest wall movement should be assessed if the heart rate does not improve. If inflation pressure is being monitored, an initial inflation pressure of 20 cm H_2_O may be effective, but 30 to 40 cm H_2_O may be required in some term infants without spontaneous ventilation. If pressure is not monitored, the minimum inflation required to achieve an increase in heart rate should be used.[52] There is insufficient evidence to recommend an optimum inflation time. In summary, assisted ventilation should be delivered at a rate of 40 to 60 breaths per minute, to promptly achieve or maintain a heart rate >100 bpm. The optimum pressure, inflation time and flow required to establish an effective *Functional Residual Capacity* (FRC) has not yet been determined.

### Devices

Effective ventilation can be achieved with a self-inflating bag, flow-inflating bag or with a T-piece.[52–54] A T-piece is a valved mechanical device, designed to control flow and limit pressure. The pop-off valves of self-inflating bags are flow-dependent, and the pressures generated may exceed the value.[55] Target inflation pressures and long inspiratory times are more consistently achieved with a T-piece, rather than with bags, although the clinical implications are not clear.[56] To provide the desired pressure, healthcare providers need more training in the use of flow-inflating bags than with self-inflating bags.[57]

Laryngeal mask airways (LMAs) that fit over the laryngeal inlet have been shown to be effective for ventilating newly born near-term and full-term infants.[58,59] There are limited data on the use of these devices in small preterm infants.[60,61] The use of the LMA can provide effective ventilation in a time frame consistent with the current resuscitation guidelines,[59,61,62] although the infants studied were not being resuscitated. A controlled trial found no clinically significant difference between the use of the LMA and endotracheal intubation when the bag-mask ventilation was unsuccessful.[58] When the bag-mask ventilation has been unsuccessful and endotracheal intubation is not feasible or is unsuccessful, the LMA may provide effective ventilation.[63–65] There is insufficient evidence to support the routine use of the LMA as the primary airway device during neonatal resuscitation, in the setting of meconium-stained amniotic fluid, when chest compressions are required, in very low birth weight infants, or for delivery of emergency intra-tracheal medications. In the case of non-successful mask ventilation, where endotracheal intubation is not possible or problematic, a laryngeal mask should be considered as a good alternative.[66,67]

### Endotracheal tube placement

Endotracheal intubation may be indicated at several points during neonatal resuscitation:

- When tracheal suctioning for meconium is required
- If bag-mask ventilation is ineffective or prolonged
- When chest compressions are performed
- When endotracheal administration of medications is desired

For special resuscitation circumstances, such as congenital diaphragmatic hernia or extremely low birth weight (< 1000 g), the timing of endotracheal intubation may depend on the skill and experience of the providers.

After endotracheal intubation and administration of intermittent positive pressure, a prompt increase in heart rate is the best indicator that the tube is in the tracheobronchial tree and is providing effective ventilation.[6–8,68] Exhaled CO_2_ detection is effective for confirmation of endotracheal tube placement in infants and very low birth weight infants.[69,70] Exhaled CO_2_ detection is useful as a quick confirmation of the accurate position of the endotracheal tube, especially when clinical judgement is uncertain.[70,71] A positive test result (detection of exhaled CO_2_) in patients with adequate cardiac output confirms placement of the endotracheal tube within the trachea, whereas, a negative test result (i.e., no CO_2_ detected) strongly suggests oesophageal intubation.[68–71] Other clinical indicators of correct endotracheal tube placement are visual assessment during intubation, condensed humidified gas during exhalation, the presence or absence of chest movement and the confirmatory method after intubation, if the heart rate remains low and is not rising. These methods have to be systematically evaluated in neonates.

### Chest compressions

Chest compressions are indicated for a heart rate that is < 60 bpm despite adequate ventilation with supplementary oxygen for 30 seconds. As ventilation is the most effective action in neonatal resuscitation and because chest compressions are likely to compete with effective ventilation, rescuers should ensure that assisted ventilation is being delivered optimally before starting chest compressions. Compressions should be delivered on the lower third of the sternum,[72,73] to a depth of approximately one-third of the anterior–posterior diameter of the chest.

**Figure 2:**
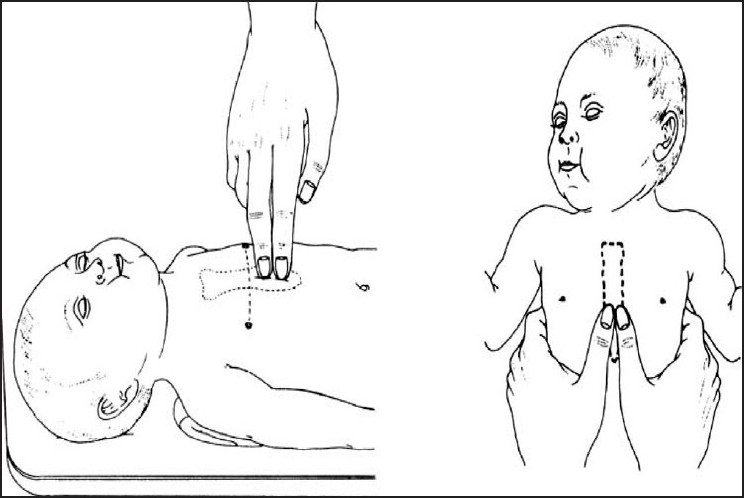
Two methods of chest compression (Neonatal Resuscitation Guidelines, Circulation, 2005)

Two techniques have been described [Figure 2]:

- Compression with two thumbs with fingers encircling the chest and supporting the back.[72,73]
- Compression with two fingers with a second hand supporting the back.

The two-thumbs-encircling hands technique may generate higher peak systolic and coronary perfusion pressure than the two-finger technique.[74,75] The two-thumb-encircling hands technique is recommended in newly born infants. However, the two-finger technique may be preferable when access to the umbilicus is required during insertion of an umbilical catheter.

Compressions and ventilations should be coordinated to avoid simultaneous delivery. The chest should be permitted to fully re-expand during relaxation, but the rescuer’s thumbs should not leave the chest. There should be a 3:1 ratio of compressions to ventilations with 90 compressions and 30 breaths to achieve120 events per minute, to maximize ventilation at an achievable rate.[76] Thus, each event will be allotted approximately second. However, chest compressions are only effective if the lungs have first been successfully aerated, making the quality of the breaths and compressions more important than the rate.

### Medications

Drugs are rarely indicated in resuscitation of the newly born infant.[77,78] Bradycardia is usually because of inadequate lung inflation or profound hypoxemia, and establishing adequate ventilation is the most important step to correct it.[75] However, if the heart rate remains < 60 bpm despite adequate ventilation with 100% oxygen and chest compressions, administration of epinephrine or volume expansion, or both, may be indicated. Rarely are buffers, a narcotic antagonist or vasopressors useful after resuscitation.

#### Epinephrine

Past guidelines recommended that initial doses of epinephrine be given through an endotracheal tube because the dose can be administered more quickly than through the intravenous route. Animal studies showed a positive effect of endotracheal epinephrine, using higher doses than are currently recommended.[79,80] However, the recommended doses of 0.01 or 0.03 mg/kg given endotracheally showed no effect.[81–83] Animal[82–84] and paediatric[84,85] studies show exaggerated hypertension, decreased myocardial function and worse neurologic function after administration of higher doses (0.1 mg/kg) via IV. Therefore, IV administration of 0.01 to 0.03 mg/kg per dose is the preferred route, because the intravenous route in neonates can easily be achieved by inserting an umbilical venous catheter. While access is being obtained, a higher dose (0.1 mg/kg) through the endotracheal tube may be considered, but the safety and efficacy of this practice have not been evaluated. The concentration of epinephrine for either route should be 1:10000 (0.1 mg/ml). Observational studies in children and animals show no better outcome when high intravenous dosages are used.[83–86] In addition, high intravenous dosages may increase the risk for intra-ventricular haemorrhage in preterm infants. Therefore, 0.01 – 0.03 mg/kg epinephrine is recommended via the intravenous route (via umbilical vein). The dosage can be repeated every one to three minutes.

#### Volume expansion

Volume expansion must be considered when blood loss is suspected or the infant appears to be in shock (pale skin, poor perfusion, weak pulse) and not responding to other resuscitative measures. An isotonic crystalloid rather than albumin is the solution of choice for volume expansion in the delivery room[87–89] The recommended dose is 10 ml/kg of normal saline, which can be repeated. In premature infants, giving volume expanders too rapidly must be avoided, because rapid infusions of large volumes can cause intra-ventricular haemorrhage. Emergency volume expansion may be accomplished with an isotonic crystalloid solution or O-negative red blood cells. Albumin-containing solutions are no longer the fluid for initial volume expansion.[87–89] Intraosseous access can serve as an alternative route for medications / volume expansion.

#### Naloxone

Naloxone is not a drug of choice as a part of initial resuscitative efforts in the delivery room for newborns with respiratory depression. If administration of naloxone is considered, heart rate and colour must first be restored by supporting ventilation. The preferred recommended route is IV or intra-muscular administration of naloxone. The recommended dose is 0.1 mg/kg, but no studies have examined the efficacy of this dose in newborns. Naloxone given to an infant born to an opioid-addicted mother has been associated with seizures.[90] Therefore, naloxone must be avoided in infants of mothers with opioid abuse. Naloxone is indicated in the infant for reversal of respiratory depression, secondary to maternal opioids, given four hours before delivery. Naloxone has a shorter half-life than the original maternal opioid. Therefore, the neonate must be monitored closely for recurrent apnoea or hypoventilation, and subsequent doses of naloxone may be required.

#### Glucose

Low blood glucose has been associated with an adverse neurologic outcome in a neonatal animal model of asphyxia and resuscitation.[91] Neonatal animals that were hypoglycemic at the time of an anoxic or hypoxic-ischaemic insult had larger areas of cerebral infarction or decreased survival, or both, when compared with the controls. One clinical study showed an association between hypoglycaemia and a poor neurologic outcome in perinatal asphyxia.[92]

The blood glucose concentration associated with the least brain injury after asphyxia and resuscitation cannot be defined based on the available evidence. Infants requiring resuscitation should be monitored and treated to maintain a normal glucose level; 50% Dextrose in a dose of 0.5 ml/kg may be given to correct hypoglycaemia.

#### Sodium bicarbonate

Use of sodium bicarbonate during resuscitation is controversial. It may be helpful to correct metabolic acidosis after a prolonged period of resuscitation.[81] However, it is harmful, particularly if given too early, as it mixes with acid and forms carbon dioxide. The lungs must be adequately ventilated to remove the carbon dioxide. The dose is 1 – 2 mEq / Kg / dose given as 4.2% solution (0.5 mEq/ml) at the rate of 1mEq / Kg / min.

### Induced hypothermia

Studies are conflicting. One multicenter trial did not show a difference in the number of survivors with severe disabilities when head cooling was used.[93] Another large multicenter trial, along with a smaller trial that evaluated systemic hypothermia, found a significant decrease in death or moderate disability at age 12 months and 18 months.[94–96] A rapid increase in body temperature could cause hypotension.[97] Cooling to a core temperature of < 33°C may cause arrhythmia, bleeding, thrombosis and sepsis, but studies have not reported these complications with modest hypothermia.[95,96,98] Avoidance of hyperthermia is particularly important in infants who may have had a hypoxic-ischaemic event.

There is insufficient data to recommend the routine use of modest systemic or selective cerebral hypothermia after resuscitation of infants with suspected asphyxia. Further clinical trials are needed to determine which infants benefit most and which method of cooling is most effective.

## CONCLUSION

Neonatal resuscitation contributes to a better care of newly born infants. Many important issues concerning neonatal resuscitation, have to be answered in the future, such as the effect of endotracheal suction in a meconium-stained, non-vigorous newborn, the outcome of preterm infants treated with occlusive plastic wrapping, the effect of inflation breaths with positive end-expiratory pressure on postnatal adaptation for newborns, the percentage and timing of additional oxygen in newborns not responding initially, the use of continuous positive airway pressure during neonatal resuscitation, the most efficacious intravenous dose of epinephrine in newborns with an asystole and the outcome of infants treated with hypothermia. In addition, implementation and training of the new guidelines in Neonatal Life Support Programmes will further contribute to the improvement in the care of newborn infants.
